# Deletion of KIBRA, protein expressed in kidney and brain, increases filopodial-like long dendritic spines in neocortical and hippocampal neurons *in vivo* and *in vitro*

**DOI:** 10.3389/fnana.2015.00013

**Published:** 2015-02-20

**Authors:** Anja Blanque, Daniele Repetto, Astrid Rohlmann, Johannes Brockhaus, Kerstin Duning, Hermann Pavenstädt, Ilka Wolff, Markus Missler

**Affiliations:** ^1^Institute of Anatomy and Molecular Neurobiology, Westfälische Wilhelms-UniversityMünster, Germany; ^2^Internal Medicine D, Department of Nephrology, Hypertension and Rheumatology, University Hospital MünsterMünster, Germany; ^3^Cluster of Excellence EXC 1003, Cells in Motion, CiMMünster, Germany

**Keywords:** synapse function, plasticity, cognition, Golgi-staining, electron microscopy, cell culture

## Abstract

Spines are small protrusions arising from dendrites that receive most excitatory synaptic input in the brain. Dendritic spines represent dynamic structures that undergo activity-dependent adaptations, for example, during synaptic plasticity. Alterations of spine morphology, changes of spine type ratios or density have consequently been found in paradigms of learning and memory, and accompany many neuropsychiatric disorders. Polymorphisms in the gene encoding KIBRA, a protein present in kidney and brain, are linked to memory performance and cognition in humans and mouse models. Deletion of KIBRA impairs long-term synaptic plasticity and postsynaptic receptor recycling but no information is available on the morphology of dendritic spines in null-mutant mice. Here, we directly examine the role of KIBRA in spinous synapses using knockout mice. Since KIBRA is normally highly expressed in neocortex and hippocampus at juvenile age, we analyze synapse morphology in intact tissue and in neuronal cultures from these brain regions. Quantification of different dendritic spine types in Golgi-impregnated sections and in transfected neurons coherently reveal a robust increase of filopodial-like long protrusions in the absence of KIBRA. While distribution of pre- and postsynaptic marker proteins, overall synapse ultrastructure and density of asymmetric contacts were remarkably normal, electron microscopy additionally uncovered less perforated synapses and spinules in knockout neurons. Thus, our results indicate that KIBRA is involved in the maintenance of normal ratios of spinous synapses, and may thus provide a structural correlate of altered cognitive functions when this memory-associated molecule is mutated.

## Introduction

Spines are actin-rich protrusions of the dendritic plasma membrane that play functional roles in biochemical compartmentalization, electrical filtering, integration of inputs, and plasticity of synapses (Bourne and Harris, 2008; Cingolani and Goda, 2008; Yuste, 2011). To subserve these roles, dendritic spines are dynamic structures that undergo morphological remodeling during development and in adaptation to sensory stimuli or in learning and memory (Bhatt et al., 2009; Holtmaat and Svoboda, 2009; Kasai et al., 2010; Lin and Koleske, 2010). While mature spines typically consist of a head and a neck that contain the postsynaptic signaling machinery and, if present, a spine apparatus (Kennedy and Ehlers, 2006; Harris and Weinberg, 2012), many investigations find that spine morphology and function are mutually dependent (Alvarez and Sabatini, 2007; Hotulainen and Hoogenraad, 2010; Kasai et al., 2010). For example, the rapid phase of synaptogenesis of glutamatergic contacts coincides with presence of more filopodial-like long protrusions from dendrites that represent a particularly dynamic type of spinous structure (Fiala et al., 1998; Jontes et al., 2000; Petrak et al., 2005; Kayser et al., 2008; Kwon and Sabatini, 2011). Since numerous neuropsychiatric disorders and behavioral abnormalities are also accompanied by alterations of different spine types (Blanpied and Ehlers, 2004; Lin and Koleske, 2010; Penzes et al., 2011), we elucidated the neuroanatomical properties of spinous synapses in a mouse model for KIBRA-deficiency, a molecule linked to cognition and synaptic plasticity.

KIBRA, also named *WWC1* for *WW* and *C*2 domain containing protein-1, is a cytoplasmic protein abundantly expressed in brain cerebral cortex and hippocampus, and in kidney and lung (Kremerskothen et al., 2003; Johannsen et al., 2008; Wennmann et al., 2014). KIBRA contains two amino terminal WW domains that are known to bind to PPxY motifs in target molecules, a C2 domain and a carboxyterminal-binding motif for PSD-95/Discs-large/ZO-1 (PDZ) modules (Schneider et al., 2010). Studies outside neurons suggest that KIBRA may function in endosomal vesicle sorting (Traer et al., 2007), cell migration (Duning et al., 2008; Rosse et al., 2009; Wilson et al., 2014) and establishment of cell polarity (Yoshihama et al., 2011). While these processes appear to be very different at first, a unifying aspect is that KIBRA and other members of the WWC family may act in the regulation of plasma membrane trafficking which affects cell morphology (Yoshihama et al., 2012; Wennmann et al., 2014).

In the brain, KIBRA is hypothesized to be involved in cognition because a genome-wide screening effort for candidate genes affecting human cognitive functions and memory formation has revealed a single nucleotide polymorphism (rs17070145 SNP; C→T substitution) in the ninth intron of the human KIBRA gene that is associated with superior performance in episodic memory tasks (Papassotiropoulos et al., 2006). Magnetic resonance imaging in carriers and non-carriers further suggests that such changes are due to increased hippocampal processing (Kauppi et al., 2011) and/or larger hippocampal volume (Palombo et al., 2013). A number of studies validate the association between rs17070145 SNP and cognitive performance in different cohorts (for a meta-analysis, see Milnik et al., 2012), and two exonic missense SNPs in the C2 domain of KIBRA are in complete linkage disequilibrium with rs17070145 (Duning et al., 2013). In neurons, KIBRA localizes to the postsynaptic density of synapses (Johannsen et al., 2008) and interacts with the brain-specific protein kinase Mζ (PKMζ; Büther et al., 2004; Yoshihama et al., 2009). KIBRA and PKMζ are upregulated during reference memory formation (Wang et al., 2014), and KIBRA counters the proteasomal degradation of PKMζ leading to impaired spatial memory performance (Vogt-Eisele et al., 2014). Unexpectedly, recent analyses of PKMζ null-mutant mice, expected to display a similar phenotype as KIBRA KO, did not reveal deficits in behavioral learning paradigms or in long-term potentiation (LTP) at the cellular level (Lee et al., 2013; Volk et al., 2013). Thus, a direct role of the PKMζ kinase in these processes appears unlikely. For KIBRA, in turn, some evidence exists that it actually affects memory/learning-related plasticity.

Two independent studies in null-mutant mice have recently demonstrated that KIBRA is involved in memory performance because deletion of the protein leads to decreased learning in contextual fear (Makuch et al., 2011) and spatial memory tasks (Vogt-Eisele et al., 2014). At the cellular level, these phenotypes are accompanied by impaired synaptic LTP, long-term depression (LTD) and vesicle-based turnover of postsynaptic α-amino-3-hydroxy-5-methyl-4-isoxazolepropionic acid (AMPA) receptors (Makuch et al., 2011). While these data convincingly show that KIBRA affects functional aspects of synaptic plasticity, the impact of this protein on synapse structure and dendritic spine morphology remains unknown. Here, we investigate for the first time the morphology of spinous synapses in KIBRA knockout mice. Our analysis demonstrates an increase of filopodial-like long protrusions and a reduction of perforated synapses and spinules in knockout neurons, putative structural correlates of KIBRA’s presumed role in higher cognitive functions.

## Materials and methods

### Animals

Generation of the KIBRA knockout mice used in this study was recently reported (Vogt-Eisele et al., 2014), and were maintained on a 129SV/C57BL6/N hybrid background. All experiments were performed on homozygous males and females (KO = KIBRA^−/−^) and their age-matched littermate controls (WT = KIBRA^+/+^), derived from heterozygous breeding. Animals were deeply anesthetized using isoflurane and/or decapitated, and brains removed until further use. The experimental procedures strictly followed governmental regulations of animal welfare approved by the Landesamt für Natur, Umwelt und Verbraucherschutz, North Rhine-Westphalia (license number 84-02.05.20.11.209),^1^ and were re-approved by the Institutional Animal Care and Use Committee (ZTE) of the Medical Faculty of the Westfälische Wilhelms-University, Münster, Germany. Mice were housed in a 12 h light-dark cycle in stable conditions of temperature and with access to food and water *ad libitum*.

### Morphological analysis of brain tissue

#### Modified Golgi-impregnation

Anesthetized juvenile mice (P21) were transcardially perfused with 60 ml of 0.9% sodium chloride at 37°C. After dissection, brains were incubated in filtered Golgi-Cox solution for 14 days at room temperature (RT) with one change of solution after 48 h, essentially as described (Glaser and Van der Loos, 1981; Gibb and Kolb, 1998; Dudanova et al., 2007). Brains were placed in 30% sucrose for 5 days at RT with one change of solution after 7 h, cut into 200 µm thick coronal sections, containing somatosensory cortex and hippocampus, on a vibratome (Leica VT 1000S), mounted on gelatinized microscope slides, and stored in 6% sucrose until all sections are collected. Golgi-impregnated sections were successively processed by staining slides with 12.5% ammonium hydroxide (Fluka) for 30 min, and sodium thiosulfate (Merck) for 10 min, preceded and followed by washes with dH_2_O. Sections were dehydrated in ascending ethanol series and xylene, and embedded with Entellan. Solutions, brains and sections were kept in the dark throughout the procedure.

**Figure 1:**
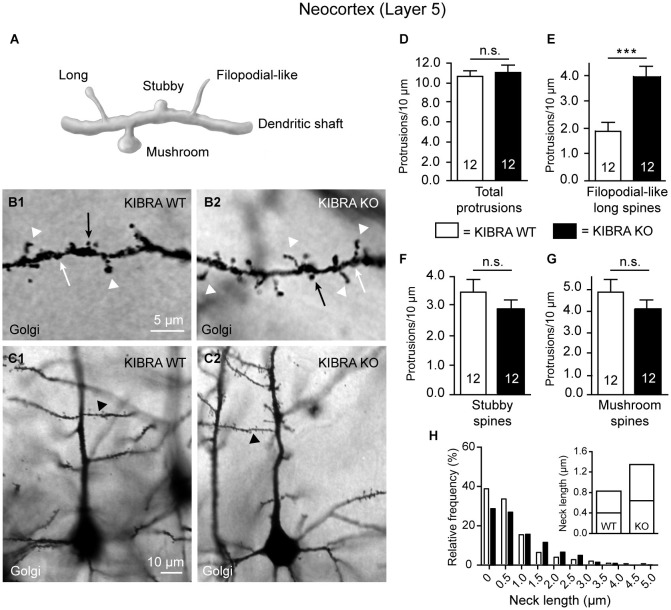
**Neocortical KIBRA-deficient neurons carry more filopodial-like long dendritic spines. (A)** Schematic overview of standard categories of dendritic protrusions used for analysis of spine densities. **(B,C)** Examples of second-order branches of apical dendrites arising from Golgi-impregnated layer 5 pyramidal neurons in the somatosensory cortex from WT and KO mice as chosen for counting of spine types (**B1,B2** show high magnification of the corresponding dendritic branch marked in (**C1,C2**) by arrowhead). Densities of all dendritic protrusions **(D)**, filopodial-like long (**E**, arrowheads in **B1,B2**), stubby (**F**, white arrows in **B1,B2**), and mushroom spines (**G**, black arrows in **B1,B2**) quantified in WT and KO samples. **(H)** Frequency distribution of neck lengths across all types of spines (bin width = 0.5 µm), inset shows median and interquartile range. Data in **(D–G)** are displayed as means ± SEM; *n* = 12 branches from 12 neurons/3 animals. Statistical analysis was done with a Mann-Whitney test (for exact median and *U*-values see Section Filopodial-Like Long Protrusions are Increased in the Neocortex of KO of the main text), and level of significance indicated as ***P < 0.001; n.s., not significant. Scale bars = 5 µm **(B1,B2)**; 10 µm **(C1,C2)**.

Golgi-stained sections were imaged with an AxioImager.Z2 microscope (Zeiss) and a CCD camera Spot Explorer (Diagnostic Instruments, Sterling Heights, USA) using the z-stack option of the imaging software (VisiView, Visitron, Germany). To examine spine distribution, the first 40–70 µm of randomly chosen first-order branches of apical dendrites from pyramidal neurons in layer 5 of the somatosensory cortex and stratum radiatum of hippocampal CA1 were acquired with a 100x oil objective and a step-width of 0.5 µm. At least 12 branches from 4 different neurons were analyzed per animal. Distance from the cell soma to the branches varied between 30–60 µm. According to their morphology, protrusions were distinguished into four categories (Figure 1A): (1) mushroom spines with a short neck (<1 µm) and a head; (2) stubby spines with a head but without a neck; (3) long spines with a long neck (>1 µm) and small heads; and (4) filopodia with no detectable head. To avoid any ambiguities from recognizing presence of a small head, groups (3) and (4) were combined for quantifications. Using Image J software (NIH, USA), the neck length was determined as the distance between the branch point from the parent dendrite to the starting point of the spine head (or to the end-point of the protrusion in case of filopodial-like long spines). For the frequency distribution of neck length across all types of spines, protrusions of up to 5 µm were included, and stubby spines without measurable neck were set to 0 and included in the first bin (0–0.5 µm length). Counting the number of protrusions that belong to the respective categories and measuring the length of analyzed branch intercepts calculated the density of spines per 10 µm dendrite.

#### Immunohistochemistry

Anesthetized juvenile mice (P21) were perfusion-fixed with 50 ml of 4% paraformaldehyde in 0.1 M phosphate buffer (PB) (37°C), and postfixed for 1 h at RT. After dissection, brains were cryoprotected in 25% sucrose/0.1 M PB overnight. For immunohistochemistry, 30 µm free-floating cryosections were treated with 1% TritonX-100 for 15 min and blocked with 50% normal goat serum (NGS)/PBS at 4°C overnight, followed by primary antibody labeling in buffer (0.1% TritonX-100, 50% NGS in PBS) at 37°C for 4 h: rb-anti-pan-synapsin (1:2000, E028, T.C. Südhof, Stanford University), rb-anti-Vgat (1:800, SynapticSystems, Göttingen, Germany), rb-anti-Vglut (1:800, SynapticSystems), rb-anti-synaptopodin (1:500, SynapticSystems). Secondary antibody goat-anti-rabbit (1:100, Covance) was applied in buffer for 30 min at 37°C, followed by rb-PAP (1:400, Sternberger) for 1 h at 37°C. Visualization was done with diaminobenzidine (0.05% w/v), H_2_O_2_ (0.005% v/v), and NiCl_2_ (0.15% w/v). Labeled sections were mounted with 0.5% gelatin, dehydrated, and embedded with Entellan. Staining was documented with an AxioImager.Z2 microscope (Zeiss) and a CCD camera Spot Explorer (Diagnostic Instruments).

#### Electron microscopy

Brain tissue from wild-type and mutant mice was embedded in epon resin (Electron Microscopy Science, EMS, Hatfield, USA). For embedding, anesthetized mice (P21) were transcardially perfused with 50 ml of 2% glutaraldehyde (Roth, Karlsruhe, Germany) and 2% paraformaldehyde (Merck, Darmstadt, Germany) in 0.1 M PB at 37°C, and postfixed at 4°C overnight. Blocks of cortical tissue were contrasted in 1% OsO_4_ for 2 h at RT. Following washes with dH_2_O and dehydrating, tissue was incubated with propylene oxide (EMS) for 45 min, infiltrated with propylene oxide/epon (1:1) for 1 h, in pure epon overnight, and hardened at 60°C for 24 h. Contrasting of thin sections from brains was done on Formvar-coated copper grids with a saturated solution of 12% uranyl acetate and lead citrate.

For ultrastructural analysis, samples were investigated with a transmission electron microscope (Libra 120, Zeiss) at 80 kV, and images taken with a 2048 × 2048 CCD camera (Tröndle, Moorenweis, Germany). For morphometry, two image series from Epon-embedded somatosensory cortex of each animal were examined at 5,000x primary magnification. Each series included images from all cortical layers, and was composed of about 17 multiple image alignment (MIA) pictures. Each MIA picture, in turn, was assembled from four adjacent images, representing an area of 100 µm^2^. MIA composition and analysis were carried out with ITEM software (Olympus Soft Imaging Solutions, Münster, Germany). Asymmetric (type 1) synapses were defined as contacts with a visible synaptic cleft, a distinct postsynaptic density and at least three synaptic vesicles. In perforated type 1 synapses, the postsynaptic density was classified as discontinuous. Spine apparatus were counted if at least 2 longitudinal tuberovesicular structures were interspersed by an electron-dense plate, and presynaptic spinules identified as objects with a double plasma membrane and a diameter <0.3 µm that were surrounded by a minimum of 3 synaptic vesicles. Synapses, spine apparatus and spinules were quantified as area densities, and the ratio of perforated to non-perforated synapses calculated. All parameters were averaged across all cortical layers and for layer 5 separately to allow direct comparison with the Golgi-Cox analysis.

### Neuronal cultures

#### Preparation and transfection

Cultures were prepared from cortex and hippocampus of WT and KIBRA KO mouse embryos (E17) as described previously (Niesmann et al., 2011; Repetto et al., 2014). Briefly, after dissection tissue was digested with 0.25% trypsin and triturated mechanically. Neurons were seeded onto poly-L-lysin-coated coverslips at low density and placed upside down above a layer of astrocytes containing N2.1 medium. 100 cells mm^−2^ were plated for transfection procedures and 50 cells mm^−2^ for antibody staining. Neurons were transfected with pMH4-SYNtdimer2-RFP (T. Oertner, Basel, Switzerland) on DIV4 by calcium phosphate transfection.

#### Immunocytochemistry

For immunocytochemistry, coverslips with primary neurons were fixed with 4% paraformaldehyde/4% sucrose for 8 min, washed with PBS, and permeabilized with 0.3% Triton-X100/PBS for 10 min. After blocking in 5% NGS/PBS for 30 min, incubation with primary antibodies followed overnight at 4°C: rb-anti-pan-Synapsin (1:500, E028, T. Südhof, Stanford University), ms-anti-PSD-95 (1:500, NeuroMab), ch-anti-MAP2 (1:10,000, Abcam), rb-anti-Cofilin1 (1:7,000, Abcam), rb-anti-protein-interacting-with-C-kinase-1 (PICK1) (1:500, Proteintech), as well as Alexa568-phalloidin (1:100, Invitrogen), all diluted in 5% NGS/PBS. After washing, cells were incubated with the following secondary antibodies: Alexa488 goat-anti-rabbit IgG, Alexa647 goat-anti-chicken IgG (Invitrogen), Cy3-conjugated goat-anti-mouse IgG (Jackson Immuno Research), diluted 1:500 in 5% NGS/PBS for 1 h at RT. After additional washings in PBS, coverslips were embedded in mounting medium (Dako).

#### Image analysis

Images of primary neurons were acquired with a 63x oil immersion objective on a Zeiss AxioImager.Z2 fluorescence microscope equipped with confocal Light Grid (Visitron System, Germany) and CCD camera Spot Explorer (Diagnostic Instruments). Alternatively, a four laser equipped VisiScope confocal Cell Explorer microscope (Zeiss and Vistron System GmbH) was used. The maximum projection of z-stacks for each image was analyzed. Spinous protrusions on cultured neurons were classified into the same categories as used for Golgi-Cox impregnated tissue (see above), and filopodial-like, long types combined for quantification. For measurements of spine size, the neck length was evaluated with ImageJ software by manually drawing a vertical line from the base of the neck, close to the dendrite, to the end of it, near the head. For the filopodial-like long type, the neck was drawn to the end of the protrusion and the threshold value was corrected according as described in (Tomasoni et al., 2013; Repetto et al., 2014). Each value shown in bar graphs represents number of protrusion per 10 µm branch length. For the frequency distribution of neck length across all types of spines, protrusions of up to 5 µm were included, and stubby spines without measurable neck were set to 0 and included in the first bin (0–0.5 µm length). For the fluorescence intensity analysis, z-stacks were taken with the same acquisition parameters in wild-type and KIBRA KO cells (e.g., exposure time, gain of the camera and light source intensity) depending on the different antibodies used. A circular Region of Interest (ROI) was drawn around each fluorescent punctae on secondary dendrites of neurons and the normalized mean fluorescence intensity was calculated with ImageJ program.

### Electrophysiological recordings

Acute brain slices containing the hippocampus of littermate wild-type and KIBRA KO mice (P21) were prepared following standard procedures. In short, mice were anesthetized with isoflurane, decapitated and brains immediately transferred into ice-cold artificial cerebrospinal fluid (ACSF) (in mM: 118 NaCl, 3 KCl, 1 NaH_2_PO_4_, 20 glucose, 1.5 CaCl_2_, 1 MgCl_2_, 25 NaHCO_3_, pH 7.3, 305 mOsmol), gassed with 95% (vol/vol) O_2_ and 5% (vol/vol) CO_2_. Horizontal slices (300 µm; Vibroslicer, Campden, UK) were incubated at 32°C in ACSF for 1.5 h and stored at RT for maximal 6 h before measurements. During recordings slices were superfused with ACSF and a stimulation electrode was placed in the stratum radiatum near the CA2/CA1 border to stimulate Schaffer collaterals. Field excitatory postsynaptic potentials (fEPSPs) were recorded with an ACSF-filled glass pipette (3–5 MW) positioned in the CA1 stratum radiatum near the pyramidal cell layer within 150–200 µm distance to the stimulation electrode. Stimuli (18–56 V, 0.1 ms) were applied every 30 s. LTP was induced after at least 15 min with constant responses with two high frequency stimulations (1 s, 100 Hz) with 30 s inter-train interval. For analysis, the slope of the first millisecond of the fEPSP was evaluated and normalized to the last 10 min before LTP induction. Representative traces were averaged from 10 consecutive recordings and stimulus artifacts were shortened for clarity.

### Statistics

Each experiment was performed at least three times on independent cell cultures and/or mice per genotype, with exact *N* values given in the figures or figure legends. Quantitative data obtained were subjected to statistical analysis by unpaired *t*-test or the nonparametric Mann-Whitney U-test for non-Gaussian distributed data sets, using Prism 6 (GraphPad Software Inc., La Jolla, CA, USA); *U*-values are given in the text, significance levels are denoted as outlined in the figure legends with *p* value < 0.05 considered significant. Mann-Whitney was chosen to compare spine numbers because some data sets did not fulfill the normality requirements according to a D’Agostino-Pearson test. All histograms in the figures display the data as means ± SEM.

## Results

Two previous studies reported the generation of deletion mouse mutants of the memory-associated molecule KIBRA by targeting exon 4/5 (Makuch et al., 2011) and exon 15 (Vogt-Eisele et al., 2014) of the *WWC1* gene. Both studies consistently demonstrated impaired learning performance by behavioral testing of homozygous mutant animals (Makuch et al., 2011; Vogt-Eisele et al., 2014). While impaired synaptic plasticity with reduced hippocampal LTP and LTD was observed in adult mice at the cellular level (Makuch et al., 2011), it remained open from these investigations if there are any structural alterations at KIBRA-deficient synapses. To answer this important question, we studied the morphology of spinous synapses in the neocortex and hippocampus of KIBRA KO mice, both in brain tissue and in neuronal cultures, because KIBRA expression is normally high in these regions (Johannsen et al., 2008).

### Alterations of spinous synapses in KIBRA-deficient mouse brain

#### Filopodial-like long protrusions are increased in the neocortex of KO

To study if deletion has an effect on the morphology of spinous contacts *in vivo*, we used KO mice (Vogt-Eisele et al., 2014) and their littermate controls at age P21 to visualize different types of dendritic spines from layer 5 pyramidal neurons in the somatosensory cortex (S1) by Golgi-impregnation of frontal sections (Figure 1). The juvenile age was chosen because abundance of KIBRA declines afterwards both at the mRNA and protein level (Johannsen et al., 2008). We distinguished between standard types of dendritic protrusions (Peters and Kaiserman-Abramof, 1970; Petrak et al., 2005; Bourne and Harris, 2008; Harris and Weinberg, 2012), i.e., (i) filopodial-like; (ii) long; (iii) mushroom; and (iv) stubby spines (Figure 1A). Spines were identified and counted on segments of first-order branches (Figure 1B) from apical dendrites (Figure 1C). To avoid any ambiguities from recognizing presence of a small head, we combined the first two types (i and ii) for quantification into one group denoted as “filopodial-like long spines”. We determined their density by a semi-automated procedure using Image J software as described in detail in the methods section. No gross anatomical abnormalities in size or branching of the dendritic tree could be detected. However, we observed an elevated number of filopodial-like long spines (arrowheads in Figures 1B1,B2) in animals lacking the KIBRA protein. Although the total density of spines along these dendrites was similar in both genotypes (Figure 1D), showing values comparable with published results obtained both from Golgi-studies and 2-photon laser-scanning microscopy (Peters and Kaiserman-Abramof, 1970; Holtmaat et al., 2005; Ballesteros-Yáñez et al., 2006; Romand et al., 2011), filopodial-like long spines from KO neurons reached about 200% of the littermate controls (Mann-Whitney statistical analysis: median spine density of WT = 1.88 and KO = 3.94, *U*-value = 14, *n* = 12 dendritic segments; *P* = 0.0004) (Figure 1E). Since filopodial-like long spines were increased, we expected to see a concomitant decrease in the remaining types of spines but we found that there was only a tendency towards lower numbers in KIBRA-deficient neocortical neurons for stubby (Mann-Whitney analysis: median density of WT = 3.52 and KO = 2.91, *U-value* = 52, *n* = 12 segments; *P* = 0.26) (Figure 1F) and mushroom spines (Mann-Whitney analysis: median density of WT = 4.39 and KO = 4.09, *U-value* = 64, *n* = 12 segments; *P* = 0.66) (Figure 1G). The increase of the number of filopodial-like long spines could represent a selective addition of particularly long protrusions or could reflect a more general tendency of spine neck length to grow longer in absence of KIBRA. We thus analyzed the frequency distribution of the neck length across all types of spines, and observed that KO spines generally outnumber WT spine in bins of longer neck length (Figure 1H). Occurrence of more spines with long necks on KO neurons is reflected by their extended interquartile range towards higher values (inset in Figure 1H). For both genotypes, the high percentage of protrusions in the shortest bin (0–0.5 µm) likely represents an overestimation of the number of stubby spines (neck length set to 0 µm) due to limited spatial resolution, as recently suggested by the application of super-resolution microscopy (stimulated emission depletion (microscopy) (STED); Tønnesen et al., 2014).

#### Ultrastructure and marker proteins of neocortical synapses

**Figure 2:**
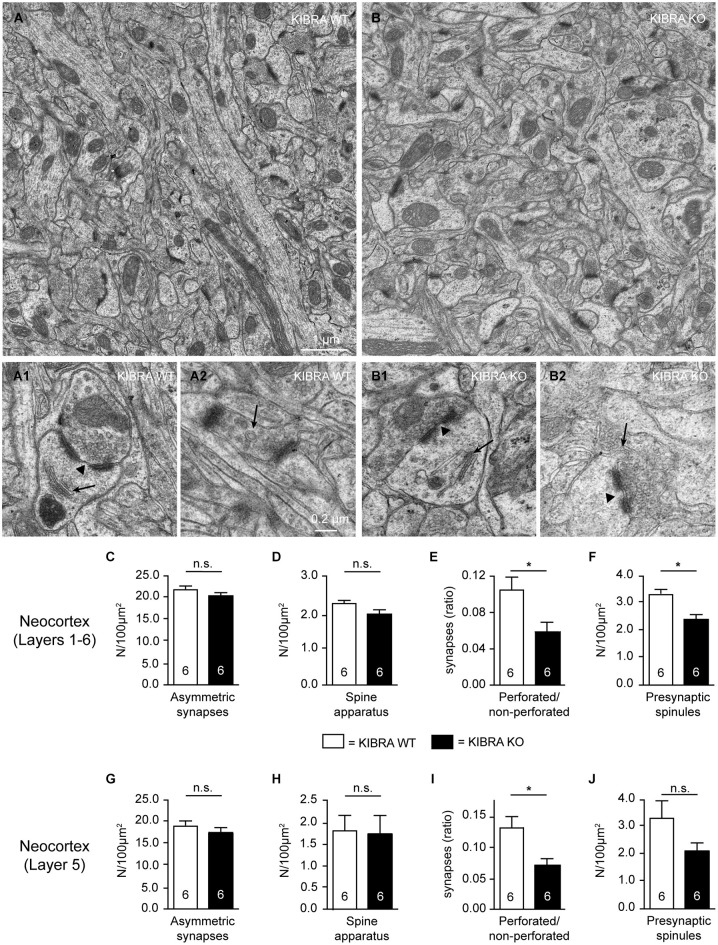
**Subtle ultrastructural defects of subpopulations of asymmetric synapses in absence of KIBRA. (A,B)** Representative electron micrographs of layer 5 neuropil from somatosensory cortex of WT and KIBRA KO mice as indicated. Prototypical asymmetric, type 1 synapses in control **(A1)** and mutant **(B1)** mouse brains, containing perforated PSDs (arrowheads) and spine apparatus (arrows). Examples of presynaptic spinules (arrows) in control **(A2)** and KO **(B2)** neurons. **(C–J)** Area density of asymmetric synapses **(C,G)**, area density of spine apparatus **(D,H)**, ratio of perforated to non-perforated asymmetric synapses **(E,I)**, and density of presynaptic spinules **(F,J)** determined across all neocortical layers **(C–F)** and for layer 5 separately **(G–J)**. Data are displayed as means ± SEM; *n* = 6 image series from 3 animals/genotype. Statistical analysis was performed by *t*-test for unpaired values, and level of significance indicated as *P < 0.05; n.s., not significant. Scale bars = 1 µm (panels **(A,B)**); 0.2 µm **(A1–B2)**.

The elongation of dendritic spines represents a remarkable finding but also raised the question if there are more deficiencies on spinous synapses in KIBRA KO brains. To assess their ultrastructure, we next performed electron microscopy in the somatosensory cortex of KIBRA mutant mice and littermate controls (Figure 2). Evaluation of tissue samples from both genotypes revealed normal organization of neuropil across all neocortical layers (Figures 2A,B for representative images from layer 5), and typical asymmetric, type 1 synaptic contacts could be identified (Figures 2A1,B1). As KIBRA KO mice suffer from altered long-term synaptic plasticity (Makuch et al., 2011), we paid particular attention to properties of structural plasticity such as perforations of postsynaptic densities (PSDs) (arrowhead in Figures 2A1,B1), spine apparatus (arrows in Figures 2A1,B1) and spinules (arrows in Figures 2A2,B2). In agreement with the light-microscopical spine analysis by Golgi-impregnation above, we found no alteration in the overall area density of asymmetric, presumably excitatory synapses averaged across all layers (WT: 21.65 ± 0.85/100 µm^2^, *n* = 6 cortical series; KO: 20.17 ± 1.12/100 µm^2^, *n* = 6 cortical series; *P* = 0.3176) (Figure 2C), or analyzed separately for layer 5 to allow direct comparison (Figure 2G). Use of electron microscopy also allowed us to directly investigate the presence and integrity of spine apparatus in KIBRA KO because these organelles, involved in the regulation of calcium or posttranslational modification of proteins, are only present in about 10–30% of spinous synapses (Sorra et al., 1998; Harris and Weinberg, 2012). Comparing their area density across layers 1–6 and separately for layer 5, we observed no quantitative differences (Figures 2D,H). However, when we determined the ratio of perforated/non-perforated synapses, an indicator of structural plasticity (Buchs and Muller, 1996; Ganeshina et al., 2004), a significant 42% reduction was seen in KIBRA mutants averaged across layers 1–6 of the somatosensory cortex (WT: 0.103 ± 0.015, *n* = 6 cortical series; KO: 0.06 ± 0.01, *n* = 6 cortical series; *P* = 0.039) and separately for layer 5 (Figures 2E,I). WT values of about 0.10–0.12 for this ratio, in turn, correspond exactly to data from other neocortical areas (Greenough et al., 1978) or the dentate gyrus (Geinisman, 2000). We also counted the number of so-called “spinules”, thin projections from spines into presynaptic boutons thought to be involved in synaptic remodeling (Sorra et al., 1998). Similar to the reduced perforations, we found a lower number of spinules in KIBRA KO brain tissue across layers 1–6 (WT: 3.27 ± 0.25/100 µm^2^, *n* = 6 cortical series; KO: 2.33 ± 0.22/100 µm^2^, *n* = 6 cortical series; *P* = 0.0172) (Figure 2F), whereas only a non-significant tendency was observed in layer 5 separately (Figure 2J), likely due to the small number of these structures. While this analysis revealed that KIBRA has specific effects on perforations and spinules of spinous synapses, but leaves their overall density and ultrastructure unscathed, native EM cannot exclude changes in the distribution of synaptic proteins.

**Figure 3:**
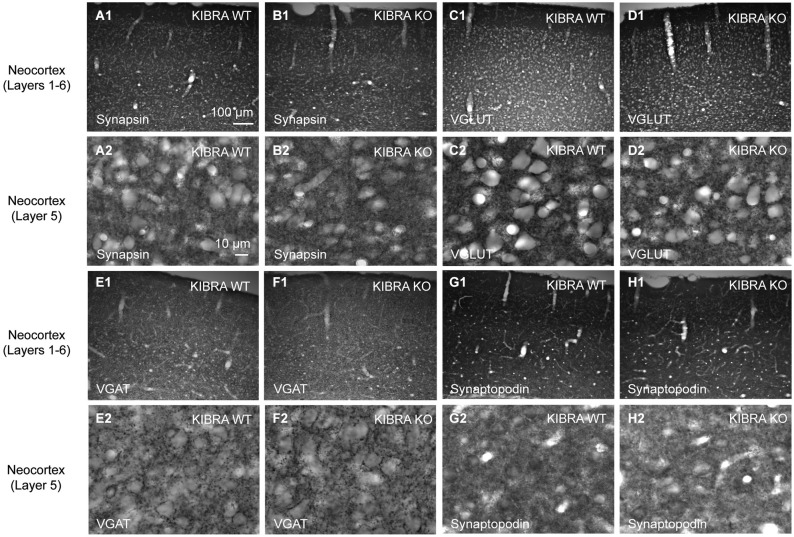
**Normal distribution of synaptic marker proteins in the somatosensory cortex of KIBRA null-mutant mice**. Representative immunohistochemical images of sagittal brain sections from WT and KIBRA KO mice as indicated. Sections were immunolabeled with antibodies against the pan-synaptic marker synapsin **(A,B)**, to VGLUT1 **(C,D)** vs. VGAT **(E,F)** to distinguish between excitatory and inhibitory terminals; and against the KIBRA-binding, spine-associated protein synaptopodin **(G,H)**. For each protein, overviews **(A1–H1)** and high magnifications from layer 5 **(A2–H2)** are shown. Series of 10–20 sections from at least 3 animals/genotype were independently assessed by two researchers for potential differences in patterns or intensity. Scale bars = 100 µm **(A1–H1)**; 10 µm **(A2–H2)**.

**Figure 4:**
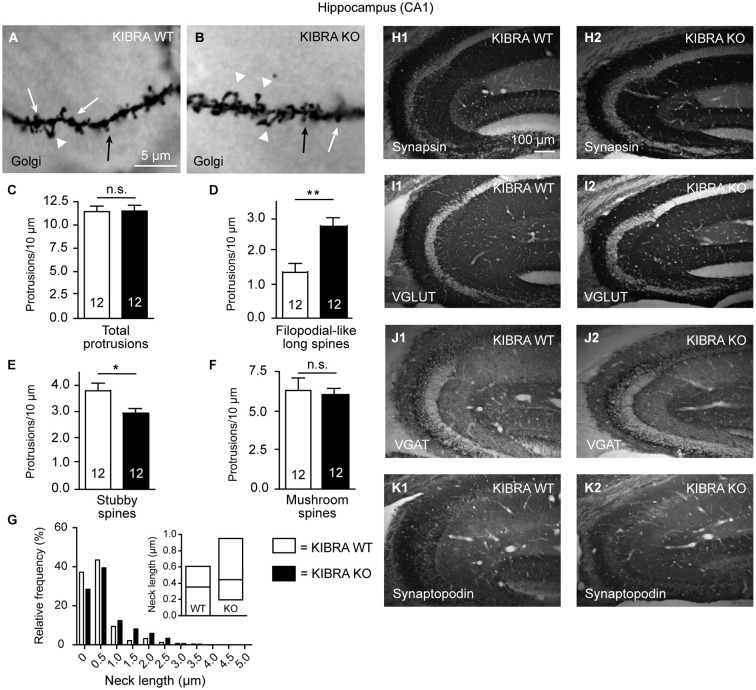
**Phenotype of spine alterations is validated in hippocampal tissue. (A,B)** Sample images of Golgi-impregnation of apical dendrite branches in the CA1 stratum radiatum from WT and KIBRA KO brains (classification of spine types as shown in Figure 1B). **(C–F)** Quantification of different spine types reveals similar increase of filopodial-like long protrusions as in the neocortical tissue (Figures 1D–G). **(G)** Frequency distribution of neck lengths across all types of spines (bin width = 0.5 µm), inset shows median and interquartile range. **(H–K)** Also similar to the somatosensory cortex, the distribution of marker proteins synapsin, VGLUT1, VGAT and synaptopodin is revealed by immunolabeled sections from WT and KO. Series of 10–20 sections from at least 3 animals/genotype were independently assessed by two researchers with no differences in pattern or intensity observed. Data in **(C–F)** are displayed as means ± SEM; *n* = 12 dendritic branches from 12 neurons/3 animals. Statistical analysis was done with a Mann-Whitney test (for exact median and *U*-values see Section Analysis of Hippocampal CA1 Confirms Increase of Filopodial-Like Long Spines but Fails to Reveal LTP Impairments in Young-Adult KIBR KO Mice of main text), and level of significance indicated as **P < 0.01; *P < 0.05; n.s., not significant. Scale bar in **(A)** = 5 µm (for **A,B**); in **(H1)** = 100 µm (for **H1–K2**).

To address the problem, we studied the distribution of relevant pre- and postsynaptic marker proteins in neocortical tissue from homozygous KIBRA KO mice compared to WT littermates (Figure 3, for more examples from hippocampus and neuronal cultures see Figures 4, 6, 7). In addition to synapsin as a ubiquitous marker of synaptic terminals (Figures 3A,B), we used antibodies against the vesicular transporters for glutamate (VGLUT1, Figures 3C,D) and GABA (VGAT, Figures 3E,F) to distinguish between putative excitatory and inhibitory synapses. Series of 10–20 sections from at least 3 animals/genotype were independently assessed by two researchers blind to the genotyping results. We observed no differences in the punctate patterns from immunolabeling the three presynaptic proteins at high magnification. In addition, no obvious change in overall intensity levels was observed in regions such as different areas and layers of neocortex and hippocampus, consistent with normal properties of release (Makuch et al., 2011). Similarly, we explored if deletion of KIBRA affected the distribution of the postsynaptic binding partner synaptopodin (Kremerskothen et al., 2005; Duning et al., 2008), a spine apparatus-associated protein with actin-bundling activity (Deller et al., 2000, 2003; Asanuma et al., 2005). However, no changes in the intensity of labeling of synaptopodin or its punctate distribution pattern at high magnification were observed in KIBRA-deficient brains (Figures 3G,H), in line with the lack of an obvious spine apparatus phenotype demonstrated above (Figure 2).

**Figure 5:**
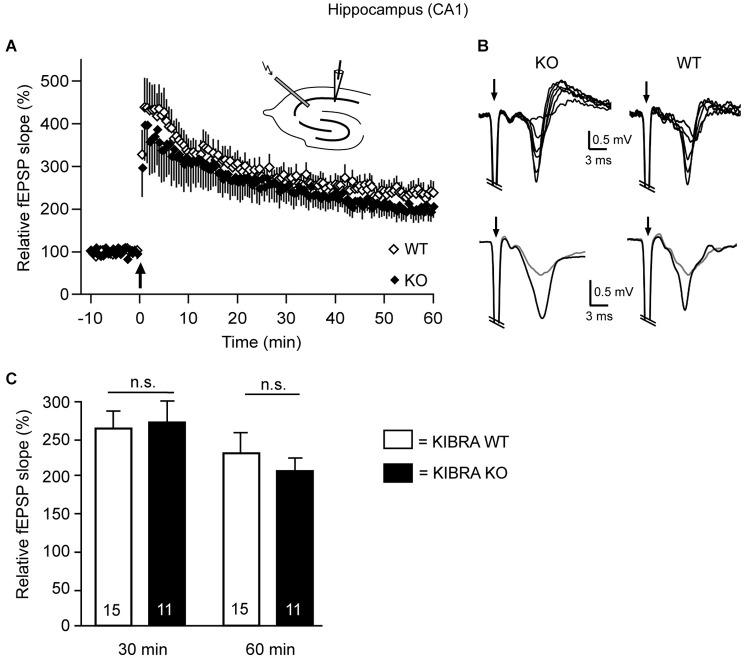
**Long-term potentiation (LTP) is unchanged in the hippocampus of juvenile KIBRA KO mice. (A)** Tetanic stimulation (arrow) in acute WT (open diamond) and KO (black diamond) sections induced a substantial and long-lasting increase in the slope of field potentials (fEPSP). Orthodrome stimulation of Schaffer collaterals with an electrode placed in the stratum radiatum consisted of 2 series (30 s interval, 1 s duration) of 100 Hz trains (0.1 ms, strength 18–56 V). fEPSPs were recorded with 0.033 Hz for at least 15 min before and 60 min after LTP induction in the stratum radiatum near the pyramidal cell layer of the CA1 region (see upper inset for approximate location of stimulation and recording electrodes). **(B)** Traces in the upper row (individual recordings) show fEPSPs in response to different stimulus intensities (20–45 V) from KO and WT slices. Lower row displays representative fEPSPs averaged from 12 recordings before (gray) and 60 min after (black) the LTP-inducing stimulus. Stimulus artifacts (arrows) are truncated. **(C)** Average potentiation of the fEPSP slope after 30 and 60 min. Data are displayed as means ± SEM, and statistics calculated by a *t*-test for unpaired values; *n* = 15 slices/4 animals (WT) and *n* = 11/4 (KO).

**Figure 6:**
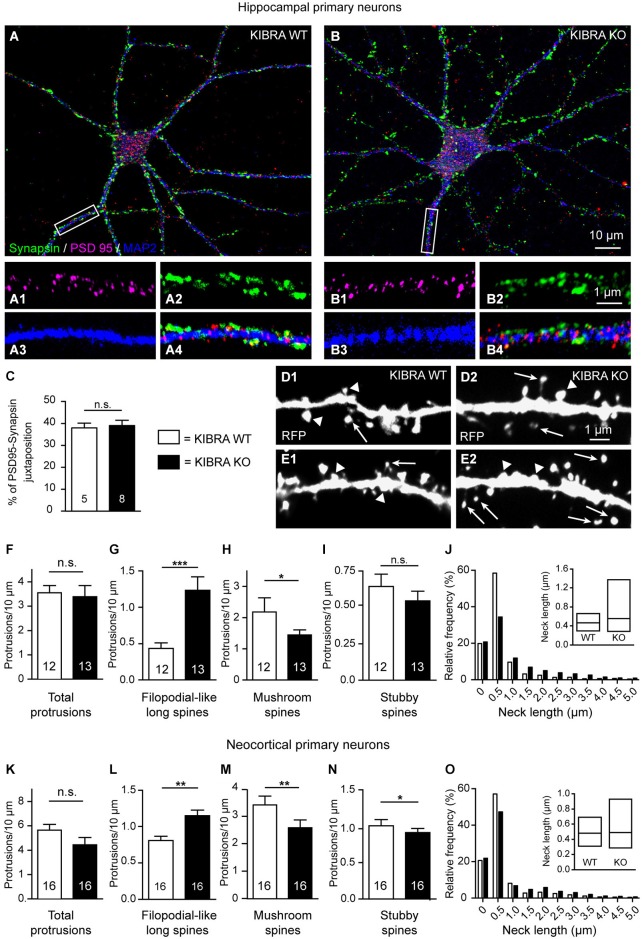
**Deletion of KIBRA disturbs dendritic spine development *in vitro* but not normal formation of synaptic contacts. (A,B)** Primary hippocampal neurons from WT and KO mice cultured at low density, and processed for immunofluorescence at DIV21. Sample images show immunolabelings with antibodies against the presynaptic marker protein synapsin (green), postsynaptic PSD95 (magenta), and dendritic MAP2 (blue). **(C)** Genotypes display normal rations of juxtaposed punctae of synapsin/PSD95, indicative of synaptic contacts, and shown at higher magnification on WT **(A1–4)** and KO **(B1–4)** dendrites corresponding to the white boxes in **(A,B)**. Scale bars = 10 µm (panels **(A,B)**); 5 µm **(A1–4,B1–4). (D,E)** Representative images of primary neurons from WT **(D1,E1)** and KO **(D2,E2)** transfected on DIV4 with monomeric red fluorescent protein (mRFP) to analyze dendritic protrusions on DIV21. Arrows indicate filopodial-like protrusions; arrowheads indicate mushroom spines in panels **(D1–2)**, and stubby spines in panels **(E1–2). (F–I)** Quantitative analysis of different types of stubby spines in panels **(E1–2). (F–I)** Quantitative analysis of different types of dendritic spines in hippocampal cultures, expressed as average number of protrusions/dendrite length. **(J)** Frequency distribution of neck lengths across all types of hippocampal spines (bin width = 0.5 µm), inset shows median and interquartile range. **(K–O)** Identical analyses for primary neurons cultured from cerebral cortex. Data in **(C,F–I,K–N)** are displayed as means ± SEM; number of analyzed cells, derived from at least 3 independent cultures/mice. Statistical analysis was done with a Mann-Whitney test (for exact median and *U*-values see Section Alterations of Dendritic Spines Studied in Primary Neurons Cultured from KIBRA-Deficient Cortex and Hippocampus of main text), and level of significance indicated as ***P < 0.001; **P < 0.01; *P < 0.05; n.s. = not significant. Scale bar = 10 µm (in **A,B**), and 1 µm for all high magnifications.

**Figure 7:**
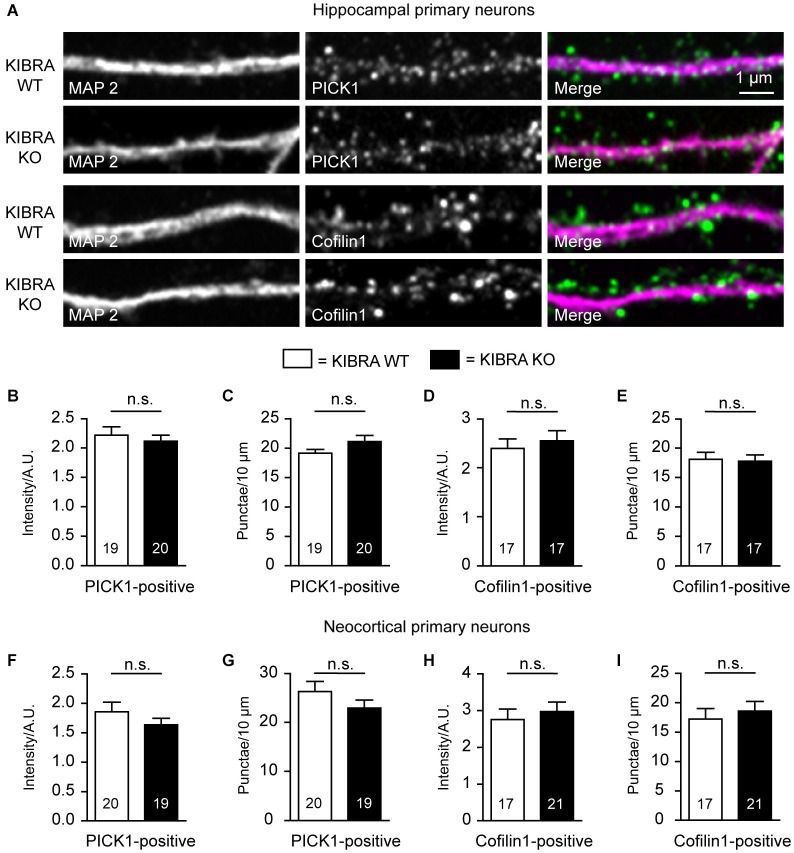
**KIBRA-deficiency has no effect on PICK1- and cofilin-positive dendritic spines in cultured neurons. (A)** Representative images of co-labelings of dendritic marker MAP2 and PICK1 or cofilin in hippocampal cultures at DIV21. **(B–E)** Quantification from image analysis of intensity and density of PICK1- **(B,C)** and cofilin-positive **(D,E)** punctae along dendrites of primary hippocampal neurons. **(F–I)** Identical analysis for primary neurons cultured from cerebral cortex. Data are means ± SEM; number of analyzed cells, derived from at least 3 independent cultures/mice, are shown in bar graphs. Statistical analysis was done by *t*-test for unpaired values, and level of significance indicated as n.s. = not significant. Scale bar = 1 µm for all images.

#### Analysis of hippocampal CA1 confirms increase of filopodial-like long spines but fails to reveal LTP impairments in young-adult KIBRA KO mice

To test the KIBRA KO phenotype of increased filopodial-like long spines in an additional brain region and to compare the structural deficiencies observed here more directly with the previously reported functional defects on Schaffer collaterals (Makuch et al., 2011), we next repeated the dendritic spine analysis in the hippocampal CA1 region (Figure 4). We distinguished the same classes of protrusions on Golgi-impregnated dendritic branches in the stratum radiatum of WT and KO frontal sections (Figures 4A,B). Counting their density, we found the total number of dendritic protrusions unchanged (Figure 4C) but again measured an almost twofold higher number of filopodial-like long spines in this brain area (Mann-Whitney analysis: median density of WT = 1.37 and KO = 2.47, *U*-value = 20, *n* = 12 segments; *P* = 0.0018) (Figure 4D). Unlike in the neocortex (Figures 1F,G), we observed a moderate concomitant reduction of stubby spines that was significant (Mann-Whitney analysis: median density of WT = 3.63 and KO = 2.83, *U-value* = 31.5, *n* = 12 segments; *P* = 0.018) (Figure 4E). Similar to the neocortical analysis, the density of mushroom-shaped protrusions only showed a tendency towards lower numbers (Mann-Whitney analysis: median density of WT = 6.71 and KO = 6.09, *U-value* = 61, *n* = 12 segments; *P* = 0.54) (Figure 4F). Also in accordance with the neocortex, more KO spines were found in bins of longer neck length (Figure 4G). To brace against the possibility of redistribution of synaptic marker proteins, in particular as the synaptopodin phenotype of reduced LTP and lack of spine apparatus was analyzed in this brain region (Deller et al., 2003; Vlachos et al., 2009), we performed the same immunohistochemistry as in the neocortex (Figure 3) but observed no alterations in the hippocampus (Figures 4H–K).

Since the earlier investigation of a KIBRA KO mouse reported impaired Schaffer collateral LTP in 3–4 months-old mutants but cautioned that the phenotype may not yet be present in juvenile animals (Makuch et al., 2011), we tested this relevant aspect by performing electrophysiology in our independently generated KO mouse line (Vogt-Eisele et al., 2014) at age P21 (Figure 5). To probe for possible modifications of LTP in the same hippocampal terminals as in the earlier study, field potentials (fEPSP) were recorded in acute horizontal sections and the initial fEPSP slope was measured before and after high frequency stimulation with two 100 Hz stimulation trains (Figures 5A,B). After 30 min, the slope was potentiated to 265 ± 25% in WT and 269 ± 32% in KO mice, and after 60 min the increase was 232 ± 26% in WT and 204 ± 33% in KO mice (Figure 5C). Our data indicate that deletion of KIBRA has no effect on the long-term synaptic plasticity in juvenile mice, confirming the observation by Huganir et al. (Makuch et al., 2011) in another mutant KIBRA line. These results are important because the structural alterations of spinous synapses identified above are clearly present at the juvenile age (Figures 1, 2, 4). Thus, we conclude that the structural phenotype cannot be compensated by expression of the WWC2 homologue as proposed for the functional LTP/LTD defect (Makuch et al., 2011; Wennmann et al., 2014).

### Alterations of dendritic spines studied in primary neurons cultured from KIBRA-deficient cortex and hippocampus

Dendritic spine formation and distribution has been frequently monitored *in vitro*, for example, to dissect the phenotype of the KIBRA interaction partner synaptopodin (Vlachos et al., 2009). Therefore, we cultured primary neurons derived from fetal hippocampi and cortices at low density for 21 days (DIV21). We first immunostained WT and KIBRA KO primary neurons from neocortex and hippocampus with antibodies against markers of presynaptic and postsynaptic compartments such as synapsin and PSD95 (Figures 6A,B) to determine if cultured neurons from both genotypes develop normal spinous contacts with expected clustering of PSD95. The pattern of immunolabeling was similar in both genotypes as assayed by properly aligned synapsin- and PSD95-positive punctae on dendrites (Figures 6A1–B4), and by quantification of the ratio of juxtaposed PSD95- and synapsin-positive punctae (Mann-Whitney analysis: median PSD95—Synapsin juxtaposition of WT = 37.48 and KO = 40.35, *U-value* = 16, *n*_WT_ = 5 cells, *n*_KO_ = 8 cells; *P* = 0.58) (Figure 6C). These results demonstrate that deletion of KIBRA did not interfere with the establishment of synapses *in vitro*, consistent with our findings in brain tissue above.

Cultures from KIBRA KO and littermate controls were then transfected with red fluorescent protein (soluble mRFP; Figures 6D,E), which fills all processes including dendritic protrusions (Niesmann et al., 2011). We identified and counted the same types of spines as in the neocortex and hippocampal CA1 of brain tissue (Figures 1, 4), and found that they developed phenotypically normal. However, we observed more filopodial-like long spines on KIBRA-mutant hippocampal neurons compared to controls, whereas the overall number of protrusion was comparable (Figures 6F,G), similar to the Golgi-impregnated samples from brain tissue analyzed above. KIBRA KO dendrites carried a 220% higher number of filopodial-like long spines per 10 µm dendrite length on DIV21 (Mann-Whitney analysis: median density of WT = 0.41 and KO = 0.93, *U*-value = 17, *n*_WT_ = 12 neurons/3 cultures, *n*_KO_ = 13/3; *P* = 0.004). Confirming the tendency suspected in Golgi-impregnation of neocortical brain tissue, we observed *in vitro* a barely significant decrease in the density of more mature, mushroom spines (Mann-Whitney analysis: median density of WT = 2.18 and KO = 1.52, *U-value* = 39, *n*_WT_ = 12 neurons/3 cultures, *n*_KO_ = 13/3; *P* = 0.0344) (Figure 6H), explaining the unchanged total number of spinous protrusions. Stubby spines appeared not significantly altered in the hippocampal neurons *in vitro* (Mann-Whitney analysis: median density of WT = 0.60 and KO = 0.49, *U-value* = 55, *n*_WT_ = 12 neurons/3 cultures, *n*_KO_ = 13/3; *P* = 0.223) (Figure 6I), albeit the data display the same tendency that existed in hippocampal tissue (Figure 4E). As in the Golgi-impregnated tissue, distribution of neck length measurements of cultured neurons revealed the generally longer protrusions in absence of KIBRA (Figure 6J). Strikingly, a similar analysis of cultured neurons from cerebral cortex revealed an almost identical quantitative result for all spine types analyzed: filopodial-long spines density was increased (Mann-Whitney analysis: median density of WT = 0.79 and KO = 1.12, *U-value* = 47, *n*_WT-KO_ = 16 neurons/3 cultures; *P* = 0.001), and the number of mature mushroom spines reduced (Mann-Whitney analysis: median density of WT = 3.56 and KO = 2.19, *U-value* = 52, *n*_WT-KO_ = 16 neurons/3 cultures; *P* = 0.003) (Figures 6K–O). These converging data from neocortical and hippocampal neurons suggest that the increased number of filopodial-long spines in absence of KIBRA represents a highly valid and reliable result.

Finally, we took advantage of the superior visibility of neurons in primary cultures to study the localization and distribution of candidate molecules that might mediate the role of KIBRA in dendritic spine formation, notably PICK1, a recently identified binding partner of KIBRA (Makuch et al., 2011), and cofilin/ADF, an actin-depolymerizing protein (Huang et al., 2006). However, in co-immunolabelings of dendritic MAP2 with PICK1 and cofilin (Figure 7A), we observed no quantitative changes in the density or the intensity of PICK1- or cofilin1-positive punctae in DIV21 cultures of neurons from hippocampus (Figures 7B–E) and cortex (Figures 7F–I). Although we cannot exclude more subtle effects, for example on the turnover rates of actin in subpopulation of spines, no differences were observed in the overall distribution of F-actin using cultures counterstained with phalloidin (data not shown).

## Discussion

This study demonstrates that KIBRA, a molecule previously linked to memory performance in humans (Papassotiropoulos et al., 2006; Milnik et al., 2012), is required for normal ratios of dendritic spine subpopulations because mice lacking KIBRA show a significant augmentation in filopodial-like long protrusions. Our results appear very reliable as we could identify the same phenotype in two brain regions (somatosensory cortex, hippocampal CA1) and by two independent experimental strategies, analyzing (i) intact tissue by Golgi-impregnation; and (ii) primary neurons in cultures by transfection.

### Functional implications of the alterations at spinous synapses

The key observation of our study is that deletion of KIBRA leads to a more than twofold increase in the number of filopodial-like long spines, at least partially at the expense of stubby and mushroom variants. Such a large change in dendritic spine types is likely to be physiologically meaningful because structure-function correlations have been proposed for this neuronal compartment (for recent reviews, see Bourne and Harris, 2008; Kasai et al., 2010; Rochefort and Konnerth, 2012). Thus, a phenotype as reported here for KIBRA KO mice can be interpreted in at least two different directions:

First, increased numbers of filopodial-like long protrusions can result from delayed maturation. During development, filopodia serve as pathfinding structures that may initiate or mediate stable contact formation and spinogenesis (Cooper and Smith, 1992; Fiala et al., 1998). This process appears to be restricted to excitatory contacts as inhibitory synapses do not develop from filopodial-like protrusions but via direct contacts between axons and dendrites (Lohmann and Bonhoeffer, 2008; Wierenga et al., 2008). For excitatory terminals, however, it was observed that a subpopulation of filopodial-like long protrusions undergoes direct transition into mature spines (Ziv and Smith, 1996; Lohmann and Bonhoeffer, 2008; Yoshihara et al., 2009). This transition can be disturbed by abnormalities during development, such as fetal alcohol syndrome (Ferrer and Galofré, 1987), leading to mental retardation. Consistently, dendrites from mentally retarded patients often appear to be covered with dendritic filopodia instead of spines (Marin-Padilla, 1972; Irwin et al., 2001), a phenotype reflected by corresponding mouse models, for example, of Fragile-X syndrome (Irwin et al., 2001). The decrease of the ratio between perforated and non-perforated synapses, also part of the structural phenotype of KIBRA mutants discovered in our study, supports such a view of developmental delay as the prevalence of perforated synapses generally increases during development (Sorra et al., 1998) and with sensory experience (Greenough et al., 1978). Expression profiles of KIBRA in humans and in rat brains also emphasize a prominent role during development because mRNA levels in the rodent cortex and hippocampal formation are high from the second postnatal day till juvenile ages, when transcript levels start to decline (Johannsen et al., 2008). The same study demonstrated that protein levels of KIBRA closely follow the down-regulation towards adult ages in most areas investigated (Johannsen et al., 2008). It can not be excluded, however, that conditions exists under which KIBRA expression may be induced to higher levels, possibly similar to its association partner dendrin in spines (Kremerskothen et al., 2003), which responds to acute nicotine exposure (Schochet et al., 2008).

Second, increased numbers of filopodial-like long protrusions can be a sign of structural synaptic plasticity. Alterations in neuronal activity and spine morphology are mutually dependent properties of the brain’s ability to adapt to novel stimuli. Although there is no simple correlation between synaptic release and spine numbers (Segal, 2010), it is widely agreed that reduced synaptic activity converts mature spines into filopodia or re-initiates the formation of filopodial-long spines in an attempt to compensate for the loss of synaptic input (Abe et al., 2004; Petrak et al., 2005). Blocking synaptic transmission thus causes an increase in the density and length of dendritic filopodial-long protrusions (Portera-Cailliau et al., 2003), and their motility translates into the ability to grow new spines and synapses (Kayser et al., 2008). In addition, spine morphology can be regulated by different types of afferent input. For example, cortical and thalamic afferents onto the same dendrite in the lateral nucleus of amygdala were distinguished by spine structure and function: thalamic inputs terminate on mushroom spines and show larger Ca^2+^ transients than cortical inputs that make contacts on long spines (Humeau et al., 2005). Filopodial-like long spines have been proposed as learning spines because formation of filopodia is observed within minutes of LTP-inducing stimulation (Nagerl et al., 2007) and LTP/LTD exert an effect on their conversion to mushroom shapes (Bourne and Harris, 2007). Another study has reported an increase in filopodial-like long dendritic spines following tetanic potentiation with a concomitant decrease in synapses on stubby-shaped spines (Popov et al., 2004). Moreover, tetanic potentiation caused changes in volume and area density of perforated PSDs (Popov et al., 2004), others observed more perforated synapses after LTP induction (Buchs and Muller, 1996; Toni et al., 1999). Since perforated PSDs contain more AMPAR (Ganeshina et al., 2004) and spine morphology appears to correlate with the number of AMPAR (Matsuzaki et al., 2004), such structural modifications can support synaptic enhancement during the early LTP phase. Finally, the idea of a causal link between spine morphology and function has also received support from studies of Ca^2+^ dynamics because the spine neck modulates postsynaptic Ca^2+^ signals (Yuste et al., 2000) and long spines were shown to attenuate synaptic potentials between the spine head and the parent dendrite (Araya et al., 2006). Smaller spines with longer/thinner necks exhibited larger increases in Ca^2+^ concentration, facilitating LTP induction by NMDAR activation (Noguchi et al., 2005). Longer spines, in turn, insert less AMPAR subunits in their heads than shorter ones (Korkotian and Segal, 2007).

Previous analyses of KIBRA KO mice have revealed functional deficiencies related to these processes: adult KIBRA mutants show impaired cellular forms of memory because LTP and LTD are reduced at hippocampal Schaffer collaterals and AMPAR trafficking into the postsynaptic membrane is defective (Makuch et al., 2011). The decrease of the ratio between perforated and non-perforated synapses and the reduced spinules shown here may reflect such an impairment to express normal LTP and to deliver functional AMPAR (Makuch et al., 2011). However, the study by Huganir et al. did not observe any LTP or LTD impairments in juvenile KO mice (Makuch et al., 2011), which they explained by a compensatory up-regulation of the KIBRA homologue WWC2 that diminishes by adulthood. As we discovered our structural KO phenotype of spinous synapses in mice of the age of P21, we had to re-investigate LTP expression at hippocampal CA1 Schaffer collaterals in this independently generated KIBRA KO mouse line (Vogt-Eisele et al., 2014). We here confirmed the previous finding that input/output relations and long-term plasticity appear normal in 3-week-old KO. It cannot be excluded based on our limited number of electrophysiological experiments that more subtle functional deficiencies exist that would show with more sensitive methods or different protocols, for example, by using glutamate uncaging or probing short-term plasticity. However, if confirmed, the inapparent functional defects at excitatory spinous synapses in juvenile KIBRA mutants could have an interesting implication: if neuroanatomical changes are present earlier than functional impairments, the structural alterations would represent the predominant phenotype that cannot be compensated by other KIBRA homologues (in particular WWC2). An alternative explanation for the absence of strong functional phenotypes, in particular in young neurons, could be that KIBRA has a more important role in homeostatic plasticity by contributing to synaptic scaling, a mechanism that stabilizes circuit activity within a target range (Turrigiano, 2012). In fact, the KIBRA binding partner synaptopodin was recently linked to such homeostatic functions in a partial deafferentation paradigm (Vlachos et al., 2013). Clearly, more detailed investigations of normal synapse physiology and synaptic homeostasis in KIBRA KO mice are required to distinguish between these interpretations.

### Candidates mediating the altered dendritic spine phenotype

Mechanisms that might lead to increased filopodial-like long protrusions as found in our study likely involve the actin cytoskeleton. These mechanisms are very difficult to dissect because numerous signaling pathways converge to regulate the actin cytoskeleton-dependent spine extension (Cingolani and Goda, 2008; Fortin et al., 2012). In addition, many actin-nucleating and –severing molecules act in concert (Fortin et al., 2012), and such regulations can even vary in different spine types (Korobova and Svitkina, 2010).

Actin is highly enriched at the PSD, where it clusters AMPAR and NMDAR by interacting with scaffolding molecules (Kuriu et al., 2006), and depolymerization of F-actin is able to disperse these two glutamate receptors (Allison et al., 1998). Consequently, long-term plasticity is associated with a rapid reorganization of the spine actin cytoskeleton because LTP or LTD induction shift the G-actin/F-actin ratio into opposite directions (Okamoto et al., 2004). As actin-regulating molecules can block both structural spine plasticity and functional LTP (Matsuzaki et al., 2004), we studied the distribution of cofilin, an actin-depolymerizing protein (Huang et al., 2006) with a localization at the postsynaptic density (Racz and Weinberg, 2006), similar to KIBRA. Functional LTP and LTD defects, observed also in adult KIBRA KO mice (Makuch et al., 2011), have been reported to lead to spine alterations mediated by cofilin (Zhou et al., 2004; Chen et al., 2007). In addition, spine shapes are altered in KO mice of the cofilin-phosphorylating kinase LIM (Meng et al., 2002), and leucine-rich repeat kinase 2 affects phosphorylation of cofilin indirectly via the PKA regulatory subunit IIβ (Parisiadou et al., 2014). However, in our study no differences were observed in the overall distribution and intensity of cofilin1-labeled dendritic spines in hippocampal and cortical cultures. More subtle effects on its phosphorylation cannot be excluded at present as available reagents failed to reliably show phospho-cofilin in dendritic spines of our cultures.

KIBRA interacts directly with synaptopodin, an actin-bundling protein enriched in dendritic spines (Mundel et al., 1997; Deller et al., 2000; Asanuma et al., 2005; Kremerskothen et al., 2005; Korkotian and Segal, 2007). Silencing of synaptopodin has been shown to cause formation of filopodia from podocytes (Asanuma et al., 2006), and its deletion in mice leads to reduction of LTP and an impairment of learning behavior (Deller et al., 2003), at least partially mimicking the KIBRA KO phenotype (Makuch et al., 2011; Vogt-Eisele et al., 2014). Since synaptopodin-deficient animals completely lack spine apparatus (Deller et al., 2003), we investigated their presence in our KIBRA mutants. However, we found a normal distribution of these spine organelles in electron microscopy and unchanged immunohistochemistry for synaptopodin in KIBRA KO, and synaptopodin mutants in turn show normal spine type distribution (Deller et al., 2003). Therefore, we conclude that the association between KIBRA and synaptopodin does at least not critically determine the phenotype reported in this study.

More recently, PICK1 was revealed as an additional binding partner of the multi-domain molecule KIBRA (Makuch et al., 2011). Through this link KIBRA could be involved in the regulation of actin because PICK1 binds to F-actin and affects the actin cytoskeleton in immature neurons (Rocca et al., 2008). PICK1 is also known to interact with AMPAR and regulates the sorting of AMPAR subunits from early endosomes to a recycling compartment (Shepherd and Huganir, 2007). This pathway is facilitated by association of PICK1 with PI(3)P-containing membranes on early endosomes (Citri et al., 2010; Jaafari et al., 2012). Interruption of this pathway by deletion of KIBRA showed that KIBRA is actually able to regulate the recycling of vesicle-associated AMPAR (Makuch et al., 2011). Consistently, reinsertion of AMPA receptors into the postsynaptic membrane is accelerated by knockdown of KIBRA in cultured neurons (Makuch et al., 2011). Our previous biochemical and structural analyses demonstrated that the KIBRA C2 domain has lipid binding capacity with a preference towards PI(3)P that is altered by certain polymorphisms (Duning et al., 2013). It can be hypothesized that KIBRA may act as a docking station for PICK1/AMPAR on PI(3)P-containing endosomes. We therefore probed the consequence of the deletion of KIBRA on PICK1 distribution in our neuronal cultures but failed to detect overt differences in density or intensity of PICK1-positive spines. Since PICK1 is not functioning alone but its action includes additional binding partners, for example the actin-nucleating Arp2/3 complex that is inhibited by PICK1 (Rocca et al., 2008, 2013), future research will need to clarify a putative role of the KIBRA-PICK1 interaction in regulating the actin cytoskeleton. Similarly, we also did not see clear differences between control and KO cultures in the distribution of F-actin itself. However, it should be noted that these experiments do not exclude effects of KIBRA on the actin cytoskeleton, for example on the dynamics or turnover rates of actin, in particular if subpopulations of spines are impacted.

## Conclusions

Our results presented in this study are highly relevant because they report the first structural defect in a mouse model of KIBRA, a molecule related to memory-performance. The neuroanatomical data appear reliable due to investigation of two brain regions and analysis by independent experimental strategies. We propose that the morphological alterations at spinous synapses discovered in KIBRA KO mice accompany or even precede functional and behavioral abnormalities, emphasizing their importance. If comparable alterations in structure and output properties of spinous synapses also underlie the symptoms in patients carrying KIBRA polymorphisms or mutations, remains an important question for future studies.

## Author contributions

All authors gave their approval of the manuscript and agree to take responsibility for the integrity of their data and the accuracy of their data analysis. Study concept and experimental design was done by MM and AR. Acquisition of data and analysis were performed by AB, DR, AR, JB and IW. Interpretation of data was done by AB, DR, AR, JB and MM. Material was provided by KD and HP. The article was drafted by MM and critically revised by all authors.

## Conflict of interest statement

The authors declare that the research was conducted in the absence of any commercial or financial relationships that could be construed as a potential conflict of interest.

## Footnotes

^1^www.lanuv.nrw.de
